# Ameliorating effects of gypenosides on chronic stress-induced anxiety disorders in mice

**DOI:** 10.1186/s12906-015-0856-4

**Published:** 2015-09-14

**Authors:** Ting Ting Zhao, Keon Sung Shin, Hyun Sook Choi, Myung Koo Lee

**Affiliations:** College of Pharmacy and Research Center for Bioresource and Health, Chungbuk National University, Cheongju, Chungbuk 28644 Republic of Korea; Department of Food and Nutrition, Chungcheong University, Cheongju, Chungbuk 28171 Republic of Korea

**Keywords:** Gypenosides, Chronic stress-induced anxiety disorders, Elevated plus-maze, Dopamine, Serotonin, Corticosterone

## Abstract

**Background:**

Ethanol extract from *Gynostemma pentaphyllum* (GP) shows anti-stress and anxiolytic functions in mice, and also protects dopamine neurons in 6-hydroxydopamine-lesioned rat model of Parkinson’s disease. In addition, gypenosides (the gypenoside-enriched components of GP, GPS) have a protective effect on 1-methyl-4-phenyl-1,2,3,6-tetrahydropyridine-induced mouse model of Parkinson’s disease. In this study, the ameliorating effects of GPS on chronic stress-induced anxiety disorders in mice were investigated.

**Methods:**

Mice were orally treated with GPS (100 and 200 mg/kg) once a day for 10 days, followed by exposure to electric footshock (EF) stress (0.6 mA, 1 s every 5 s, 3 min). After the final administration of either GPS, water extract of GP (GP-WX) or ethanol extract of GP (GP-EX, positive control), the behavioral tests such as elevated plus-maze, marble burying and locomotor activity tests, and the biochemical parameters including dopamine, serotonin and corticosterone levels, and c-Fos expression were examined.

**Results:**

Treatment with GPS (100 and 200 mg/kg) increased the number of open arm entries and the time spent on open arms in elevated plus-maze which were reduced by chronic EF stress. GPS (100 and 200 mg/kg) reduced the number of marbles buried which increased by chronic EF stress. In these states, the brain levels of dopamine and serotonin decreased by chronic EF stress and they were recovered by GPS. The serum levels of corticosterone increased by chronic EF stress were also reduced by GPS (100 and 200 mg/kg). Finally, chronic EF stress-induced c-Fos expression was markedly reduced by GPS (100 and 200 mg/kg) in the brain. GPS (100 and 200 mg/kg) also showed an equivalent efficacy on anxiolytic functions, as compared with GP-EX (50 mg/kg). However, GP-WX (50 mg/kg) showed a less effect on anxiety disorders than GP-EX (50 mg/kg) and GPS (100 and 200 mg/kg).

**Conclusion:**

These results suggest that GPS (100 and 200 mg/kg) has anxiolytic effects on chronic EF stress-induced anxiety disorders by modulating dopamine and serotonin neuronal activities, c-Fos expression and corticosterone levels. GPS may serve as a phytonutrient in chronic stress-induced anxiety disorders.

## Background

Slight or acute stress and repeated long-lasting or chronic stress are characterized by the physiological changes that occur in response to novel or threatening stimuli. Chronic stress has been linked to the pathophysiology of various psychiatric disorders, including anxiety disorders and depression [1]. In the conditions of chronic stress-induced anxiety disorders and depression, the levels of dopamine and serotonin in the brain are decreased. In contrast, the serum levels of glucocorticoids such as corticosterone and cortisol are increased by various chronic stresses, and this is mediated by the hypothalamic-pituitary-adrenal axis [2]. In addition, c-Fos protein, which is an immediate early gene, is stimulated against chronic stress in various brain regions of mice and rats, especially paraventricular nuclei (PVN) of hypothalamus [3, 4].

A number of chronic stress models, including electric footshock (EF) stress, forced swimming, noise stimuli and immobilization have been employed to induce anxiety disorders [5, 6]. In the field of anxiety disorders induced by chronic stress, the elevated plus-maze and marble burying tests are widely used in response to threatening stimuli [7, 8]. Spontaneous locomotor activity can be also decreased by chronic stress [9].

The traditional medicinal herb, *Gynostemma pentaphyllum* Makino (GP, Cucurbitaceae) has been identified with about 90 dammarane-type glycoside derivatives (called Gynostemma total saponins, gypenosides or gynosaponins) and flavonoids [10]. Previously, ethanol extract from GP (GP-EX) has been reported to have anti-stress and anxiolytic functions in mice [6]. GP-EX also protects dopamine neurons in 6-hydroxydopamine-lesioned rat model of Parkinson’s disease (PD) [11]. In addition, the gypenoside-enriched components (gypenosides, GPS) have a protective effect on 1-methyl-4-phenyl-1,2,3,6-tetrahydropyridine-induced mouse model of PD [12, 13].

In this study, in order to further define the anxiolytic function of GP, the pharmacological effects of GPS, which was obtained from GP [12, 13], on chronic electric footshock (EF) stress-induced anxiety disorders in mice were investigated. In addition, the anxiolytic functions by GPS were compared with those by water extract of GP (GP-WX) and GP-EX (positive control).

## Methods

### Chemicals

Dopamine, norepinephrine, serotonin and 5-hydroxyindoleacetic acid (HIAA) were purchased from Sigma (St Louis, MO, USA). Corticosterone kit was obtained from USCN Life Sci. (E0504 m, Wuhan, China) and c-Fos antibody was from Santa Cruz Biotechnology (Santa Cruz, CA, USA). All other chemicals were of analytical grade.

### Preparation of GPS and GP extract

GPS were purchased from Ankang Dongke Maidisen Co. (purity > 98 %, determined by UV) (Xi’an, China) [12, 13]. GP was obtained from Wonkwang Food Manufacturing Co. (Geochang, Korea) and a voucher specimen of the herbal leaves of GP was deposited at the herbarium of the College of Pharmacy, Chungbuk National University (Cheongju, Korea). The air-dried leaves of GP (5 kg) were extracted twice with distilled water at 80 °C for 3 h (GP-WX) and 80 % ethanol at 25 °C for 12 h (GP-EX), respectively, and then the each extract was evaporated to dryness [6, 10, 14]. Ultimately, 390 g (7.8 %, w/w) of GP-WX and 490 g (9.8 %, w/w) of GP-EX, which were determined using high performance liquid chromatography (HPLC), were obtained.

### Experimental design

Mice (ICR, male, 20–25 g) were obtained from Samtako Co. (Osan, Korea). Mice were housed two per cage in a temperature and humidity controlled environment (12-h light/dark cycle, 23 ± 2 °C, 60 ± 2 %), with ad libitum access to standard mouse food and water. This study was approved by the Animal Ethics Committee of Chungbuk National University (Approval No., CBNU-481-12-01).

The experiment was performed by 2 separate subsets for the elevated plus-maze and marble burying tests including the biochemical analyses, and for the spontaneous locomotor activity test including the immunohistochemical analyses. Each subset contained 16 groups (8–10 animals per group): half groups were exposed to the chronic EF stress and half were not. Each eight groups were orally treated for 10 days before 4 h the exposure of EF stress as follow: control group was the group that received saline (0.9 %), each GPS-treated group was the group that received one of the different doses of GPS (30, 50, 100, 200, or 400 mg/kg), GP-WX treated group was the group that received GP-WX (50 mg/kg), and GP-EX-treated group was the group that received GP-EX (50 mg/kg). Stress was the group which was exposed to chronic EF stimuli with either saline (0.9 %), GPS, GP-WX or GP-EX for 10 days. GP-EX was used as a positive control [6].

In the first experiments, the elevated plus-maze and marble burying tests were performed on the final day (day-10) at 30 min after the exposure of EF stress. The elevated plus maze test was carried out before the marble burying test to approximate a stressor (the interval time, 15–20 min) [15]. After the behavioral tests, the mice were sacrificed to obtain brain tissues and serum for biochemical analyses. In the second experiments, the spontaneous locomotor activity test was carried out every day after 30 min of the exposure of chronic EF stress and then, the mice were also sacrificed to obtain brain tissues for immunehistochemical analyses.

### The exposure of chronic EF stress

The mice were placed individually in the electrified shock chamber for the exposure to chronic stress and they received unavoidable EF stimuli (intensity, 0.6 mA, 1 s every 5 s, periods, 3 min) at 14:00 every day for 10 days using an electric shock generator (Seil Electric Co., Taejeon, Korea) [16].

### The elevated plus-maze test

The elevated plus-maze apparatus consists of four arms: two open arms (30 × 5 cm) and two closed arms of the same size, with 16-cm-high black walls elevated 45 cm above the floor. The open and closed arms were connected via a central square (5 × 5 cm) to form a plus sign. The 5-min pre-adaptation period of 1 time for the last 3-days was conducted before the main test [17]. The number of open arm entries and the time spent on the open arms were recorded during a 5-min test period after the adaptation period of 5-min by a video camera connected to a SMART video-tracking system (Panlab S.I., Barcelona, Spain) [7].

### The marble burying test

The marble burying test was placed in a cage measuring 33 cm × 21 cm × 19 cm (l × w × h), containing bedding that was 5 cm in depth, with twenty marbles in a 4 × 5 arrangement along the same short wall of the cage. The 15-min pre-adaptation period for the last 3-days was applied before the main test [18]. Testing was conducted for a 30-min period under red light and white noise. After a 30 min, the number of marbles buried was recorded and marbles were considered to be buried if at least two thirds of their surface was covered with bedding [8].

### The spontaneous locomotor activity test

The locomotor activity was determined by a tilting-type ambulometer (Model AMB-10, O’Hara, Tokyo, Japan). Each mouse was placed in the round activity cage (20 cm in diameter and 18 cm in depth) and after an adaptation period of 10 min, the counts of horizontal movements of mice inside the cage were automatically recorded in 30 min [6].

### Measurement of dopamine, serotonin and corticosterone levels

The whole brains were dissected out and frozen in a −70 °C until analysis. For the measurement of dopamine levels, the brain tissues were homogenized in perchloric acid (300 μL, 1 M) and isoproterenol (100 pmol, internal standard), and dopamine levels were measured by an HPLC system [19]. For the measurement of serotonin levels, the brain tissues were also homogenized in trichloroacetic acid (500 μL, 0.3 M) and HIAA (300 pmol, internal standard), and serotonin levels were measured by an HPLC system [6].

Blood was collected from the heart of sacrificed mice, and was then centrifuged to obtain a serum. Corticosterone levels were assessed using an enzyme-linked immunosorbent assay kit [6].

### Immunohistochemistry of c-Fos

Mice were transcardially perfused with a paraformaldehyde solution (4 % in 0.1 M phosphate buffered saline, pH 7.4), and then the brains were removed. Coronal sections (35 μm) at the PVN regions were processed for c-Fos immunocytochemistry using a polyclonal rabbit anti-c-Fos antibody (1:500) and a biotinylated goat anti-rabbit antibody (1:1,000; Vector Laboratories, Burlingame, CA). The sections were incubated in avidin:biotinylated enzyme complex kit and exposed to 3,3′-diaminobenzidine kit for detection. c-Fos-positive cells in the PVN regions were counted using an image analysis system (Axiovision software, Carl Zeiss MicroImaging, GmbH, Jena, Germany) using a microscope (100X magnification) (Zeiss Axiophot, Carl Zeiss MicroImaging).

### Statistical analysis

Data were analyzed with one-way analysis of variance (ANOVA) followed by Tukey’s test for evaluating the dose-dependent effects, two-way ANOVA followed by Tukey’s test for evaluating the effects on chronic stress and three-way ANOVA by Tukey’s test for evaluating the effect on between the three factors, doses, un-stressed and stressed stimuli, and days. All data represented as means ± S.E.M. with *p* values < 0.05 being considered statistically significant.

## Results

### Effects of GPS on elevated plus-maze

**Fig. 1:**
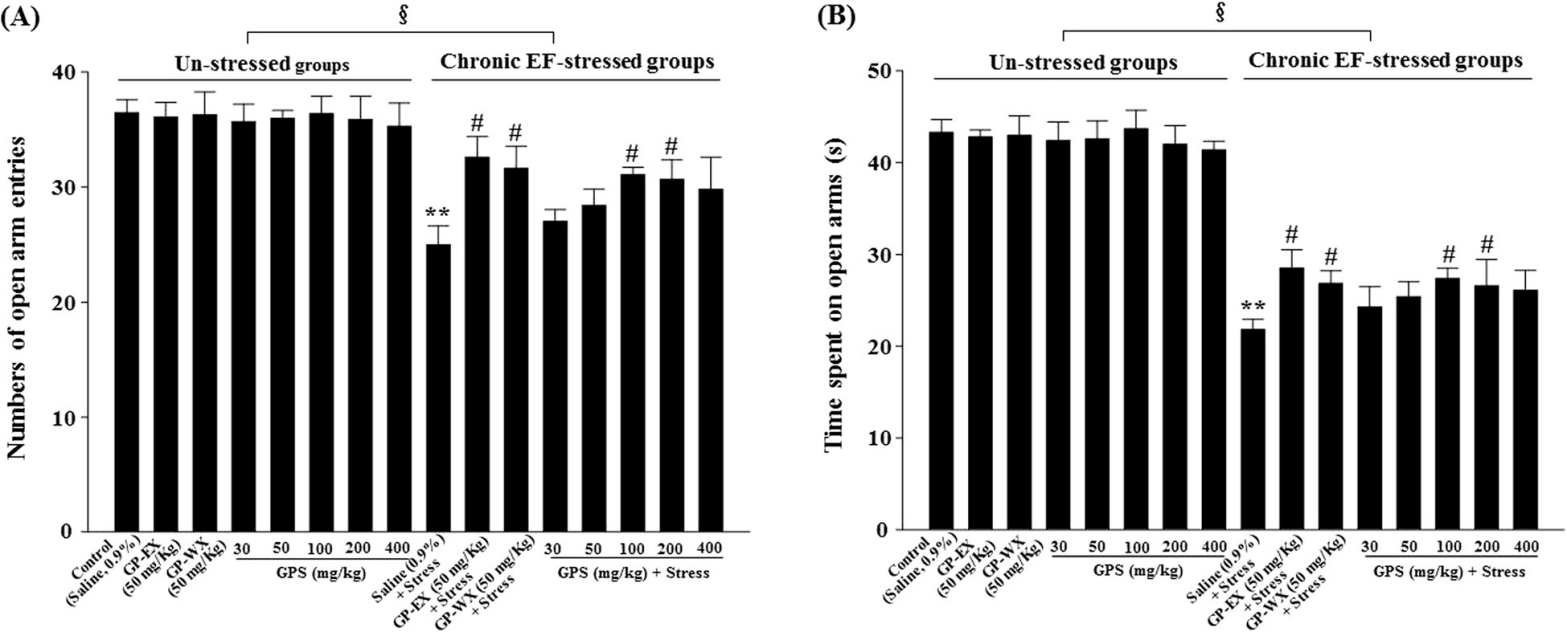
Effects of GPS on the number of open arm entries (**a**) and time spent on open arms (**b**) in the elevated plus-maze test in mice. Mice (ICR, male, 25–30 g) were orally treated with GPS (30, 50, 100, 200 and 400 mg/kg), GP-WX (50 mg/kg), GP-EX (50 mg/kg, positive control) or saline (0.9 %) once a day for 10 days. Mice were exposed to EF stimuli (intensity, 0.6 mA, 1 s every 5 s, periods, 3 min) for chronic stress for 10 days (chronic EF-stressed groups, Stress). The elevated plus-maze test was performed as described under the Methods section. The results are expressed as means ± S.E.M. for 8–10 animals per group. **p < 0.01 compared with control group; ^#^ *p* < 0.05 compared with chronic EF-stressed group (one-way ANOVA followed by Tukey’s test); ^§^ *p* < 0.05 compared with between the un-stressed groups and stressed groups (two-way ANOVA followed by Tukey’s test)

In the un-stressed groups, treatment with GPS (30, 50, 100, 200 and 400 mg/kg) once a day for 10 days did not alter both the number of open arm entries and the time spent on open arms in the elevated plus-maze test, compared with control group (Fig. 1). In contrast, the number of open arm entries and the time spent on open arms showed the significant differences between the un-stressed groups and stressed groups ((A) F = 9.411, (B) F = 16.352; *p* < 0.05) (Fig. 1). The number of open arm entries and the time spent on open arms also decreased by 31.5 % (*p* < 0.01) and 48.4 % (*p* < 0.01) by chronic EF stress, compared with control group (Fig. 1a and b). However, the number of open arm entries increased by 19.6 % (*p* < 0.05) and 18.6 % (*p* < 0.05), respectively by treatment with GPS (100 and 200 mg/kg) for 10 days, compared with chronic EF-stressed group (Fig. 1a). However, GPS (30, 50 and 400 mg/kg) did not show significant effects (7.4 %, 12.0 % and 12.7 %; *p* > 0.05) (Fig. 1a). The times spent on open arms reduced by chronic EF stress also increased by 20.4 % (*p* < 0.05) and 18.1 % (*p* < 0.05), respectively by treatment with GPS (100 and 200 mg/kg), compared with chronic EF-stressed group (Fig. 1b). However, GPS (30, 50 and 400 mg/kg) did not show significant effects (10.3 %, 14.2 % and 16.5 %; *p* > 0.05) (Fig. 1b).

The number of open arm entries and the time spent on open arms, which decreased by chronic EF stress, were also recovered by 18.8 % (*p* < 0.05) and 21.1 % (*p* < 0.05), respectively by treatment with GP-WX (50 mg/kg) for 10 days, compared with chronic EF-stressed group (Fig. 1a and b).

### Effects of GPS on marble burying

**Fig. 2:**
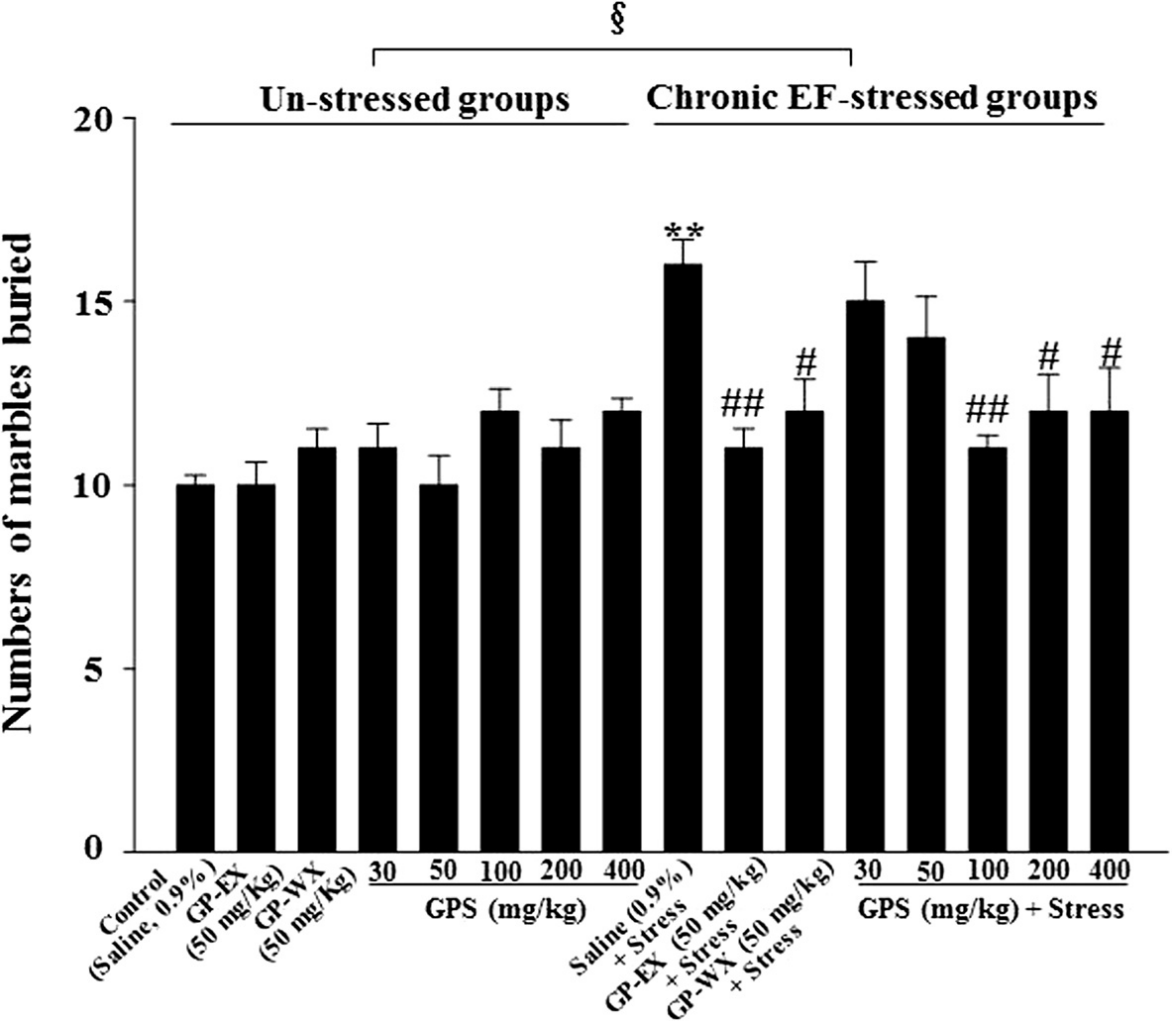
Effects of GPS on the number of marbles buried in the marble burying test in mice. Mice (ICR, male, 25–30 g) were orally treated with GPS (30, 50, 100, 200 and 400 mg/kg), GP-WX (50 mg/kg), GP-EX (50 mg/kg, positive control) or saline (0.9 %) once a day for 10 days. Mice were also exposed to EF stimuli (intensity, 0.6 mA, 1 s every 5 s, periods, 3 min) for chronic stress for 10 days (chronic EF-stressed groups, Stress). The marble burying test was performed as described under the Methods section. The results are expressed as means ± S.E.M. for 8–10 animals per group. **p < 0.01 compared with control group; ^#^ *p* < 0.05, ^##^ *p* < 0.01 compared with chronic EF-stressed group (one-way ANOVA followed by Tukey’s test); ^§^ *p* < 0.05 compared with between the un-stressed groups and stressed groups (two-way ANOVA followed by Tukey’s test)

In the un-stressed groups, treatment with GPS (30–400 mg/kg, 10 days) had no effect on the number of marbles buried. The number of marbles buried increased by 60.2 % (*p* < 0.01) by exposure to chronic EF stress, compared with control group (Fig. 2). However, the number of marbles buried of the stressed groups showed the significant differences between the un-stressed groups and stressed groups (F = 5.284, *p* < 0.05) (Fig. 2). The number of marbles buried also decreased by 31.3 % (*p* < 0.01), 25.2 % (*p* < 0.05) and 24.9 % (*p* < 0.05), respectively by treatment with GPS (100, 200 and 400 mg/kg) for 10 days, compared with chronic EF-stressed group (Fig. 2). However, GPS (30 and 50 mg/kg) did not show significant effects (6.2 % and 12.4 %; *p* > 0.05) (Fig. 2).

In addition, the number of marbles buried decreased by 25.1 % (*p* < 0.05) by treatment with GP-WX (50 mg/kg), compared with chronic EF-stressed group (Fig. 2).

### Effects of GPS on spontaneous locomotor activity

**Fig. 3:**
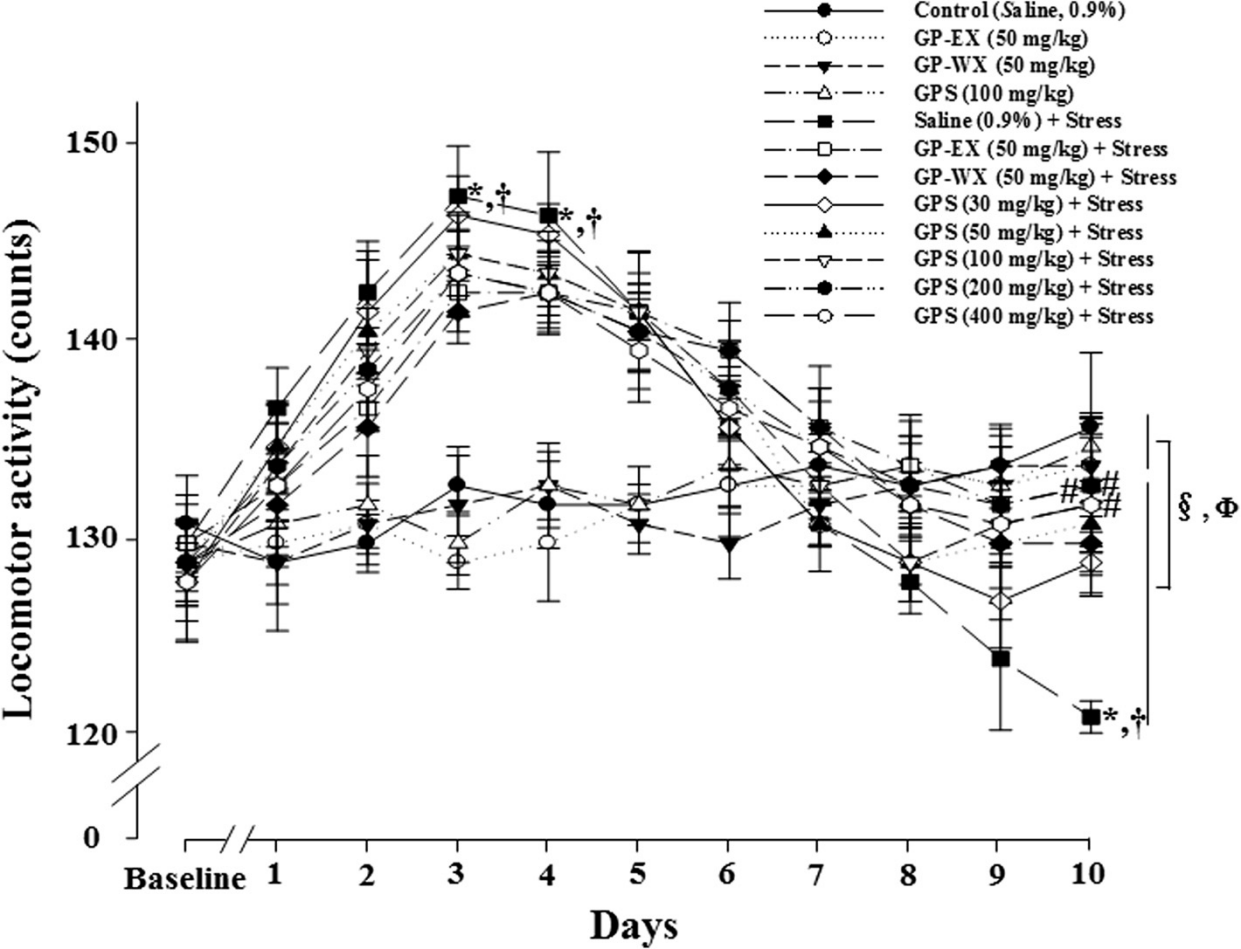
Effects of GPS on spontaneous locomotor activity after exposure to chronic EF stress in mice. Mice (ICR, male, 25–30 g) were orally treated with GPS (30, 50, 100, 200 and 400 mg/kg), GP-WX (50 mg/kg), GP-EX (50 mg/kg, positive control) or saline (0.9 %) once a day for 10 days. Mice were also exposed to EF (intensity, 0.6 mA, 1 s every 5 s, periods, 3 min) for chronic stress for 10 days (chronic EF-stressed groups, Stress). Spontaneous locomotor activity was performed as described under the Methods section. The results are expressed as means ± S.E.M. for 8–10 animals per groups. *p < 0.05 compared with control group at each day; ^#^ *p* < 0.05 compared with chronic EF-stressed group at each day); ^†^ *p* < 0.05 compared with baseline (one-way ANOVA followed by Tukey’s test); ^§^ *p* < 0.05 compared with between the un-stressed groups and stressed groups (two-way ANOVA followed by Tukey’s test); ^Φ^ *p* < 0.05 compared with day-1 group between the three factors, doses, un-stressed and stressed stimuli, and days (three-way ANOVA followed by Tukey’s test)

In the un-stressed groups, treatment with GPS (30–400 mg/kg, 10 days) exhibited no significant effect on the spontaneous locomotor activity. The spontaneous locomotor activities showed the significant differences between the un-stressed groups and stressed groups (stress treated with saline and stress treated with GPS 100, 200, 400 mg/kg) (F = 2.469, *p* < 0.05). GPS (100 mg/kg) was shown as a representative (Fig. 3). The spontaneous locomotor activities also showed a significant three-way interaction between factors, doses, un-stressed and stressed stimuli, and days (day-1 and day-10) (F = 2.041, *p* < 0.05) (Fig. 3).

In the stressed groups, the spontaneous locomotor activities showed biphasic results. The spontaneous locomotor activities by exposure to acute EF stress for 2–4 days increased by 16.8 % (*p* < 0.05) (Fig. 3), compared with control group and baseline. However, after exposure to chronic EF stress for 10 days, spontaneous locomotor activity decreased by 18.1 % (*p* < 0.05), compared with control group, which was increased 15.4 (*p* < 0.05) and 15.8 % (*p* < 0.05), respectively by GPS (100 and 200 mg/kg), compared with chronic EF-stressed group (Fig. 3). However, GPS (30, 50 and 400 mg/kg) did not show significant effects (8.7 %, 9.4 % and 12.8 %; *p* > 0.05) (Fig. 3).

Treatment with GP-WX (50 mg/kg) also increased spontaneous locomotor activity by 15.0 % (*p* > 0.05), compared with chronic EF-stressed group (Fig. 3).

### Effects of GPS on the levels of dopamine and serotonin in the brain

In the un-stressed groups, treatment with GPS (30–400 mg/kg) for 10 days did not alter the levels of dopamine and serotonin in the brain, compared with control group (GPS at 100 mg/kg was shown as a representative) (Table 1). Dopamine levels significantly decreased by 26.6 % (*p* < 0.05) after being exposed to EF stress for 10 days, compared with control group, and they increased by 18.0 % (*p* < 0.05) and 16.6 % (*p* < 0.05), respectively by treatment with GPS (100 and 200 mg/kg), compared with chronic EF-stressed group (Table 1). However, GPS (30, 50 and 400 mg/kg) did not show significant effects (8.4 %, 11.4 % and 11.9 %; *p* > 0.05) (Table 1). The levels of dopamine (F = 6.233, *p* < 0.05) and serotonin (F = 8.865, *p* < 0.05) of the stressed groups also showed the significant differences between the un-stressed groups and stressed groups (Table 1). In addition, dopamine levels increased by 19.1 % (*p* < 0.05) by GP-WX (50 mg/kg), compared with chronic EF-stressed group (Table 1).

**Table 1:**
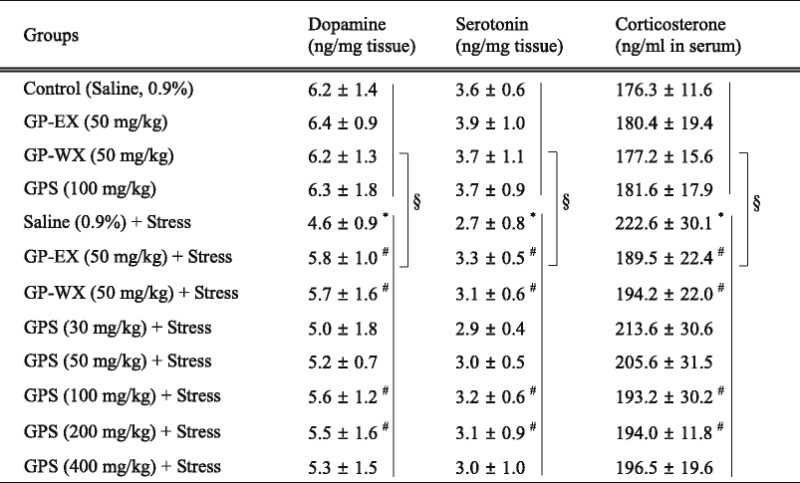
Effects of GPS on the levels of dopamine, serotonin and corticosterone

Mice (ICR, male, 20–25 g) were orally treated with GPS (30–400 mg/kg), GP-WX (50 mg/kg), GP-EX (50 mg/kg, positive control) or saline (0.9 %) once a day for 10 days. Mice were also exposed to EF stimuli (intensity, 0.6 mA, 1 s every 5 s, periods, 3 min) for chronic stress for 10 days (chronic EF-stressed group, Stress). The brains were removed, and the levels of dopamine and serotonin in brain were determined by an HPLC method. The blood samples were collected on the last day of the behavioral tests, and the levels of corticosterone in serum were determined by an enzyme-linked immunosorbent assay kit. GPS (100 mg/kg) was shown as a representative of the un-stressed groups. The results are expressed as means ± S.E.M. for 8–10 animals per group. *p < 0.05 compared with control group; ^#^ *p* < 0.05 compared with chronic EF-stressed group (Stress) (one-way ANOVA followed by Tukey’s test). ^§^ *p* < 0.05 compared with between the un-stressed groups and stressed groups (two-way ANOVA followed by Tukey’s test)

Serotonin levels also decreased by 25.1 % (*p* < 0.05) by chronic EF stress, compared with control group (Table 1). However, serotonin levels were reduced by chronic EF stress increased by 17.6 % (*p* < 0.05) and 15.7 % (*p* < 0.05), respectively by treatment with GPS (100 and 200 mg/kg), compared with chronic EF-stressed group (Table 1). However, GPS (30, 50 and 400 mg/kg) did not show significant effects (6.3 %, 9.1 % and 10.5 %; *p* > 0.05) (Table 1). In addition, serotonin levels increased by 16.1 % (*p* < 0.05) by treatment with GP-WX (50 mg/kg), compared with chronic EF-stressed group (Table 1).

### Effects of GPS on the levels of corticosterone in the serum

In the un-stressed groups, the levels of corticosterone in the serum were not altered by treatment with GPS (30–400 mg/kg) for 10 days, compared with control group (GPS at 100 mg/kg was shown as a representative) (Table 1). Corticosterone levels increased by 26.3 % (*p* < 0.05) by exposure to chronic EF stress for 10 days, compared with control group (Table 1). The levels of corticosterone also showed the significant differences between the un-stressed groups and stressed groups (F = 3.454, *p* < 0.05) (Table 1). However, corticosterone levels decreased by 16.1 % (*p* < 0.05) and 15.8 % (*p* < 0.05), respectively by treatment with GPS (100 and 200 mg/kg) for 10 days, compared with chronic EF-stressed group (Table 1). However, GPS (30, 50 and 400 mg/kg) did not show significant effects (6.1 %, 8.4 % and 14.1 %; *p* > 0.05) (Table 1).

The levels of corticosterone were also reduced by 15.0 % (*p* < 0.05) by treatment with GP-WX (50 mg/kg), compared with chronic EF-stressed group (Table 1).

### Effects of GPS on c-Fos-immunoreactive cells in the PVN

**Fig. 4:**
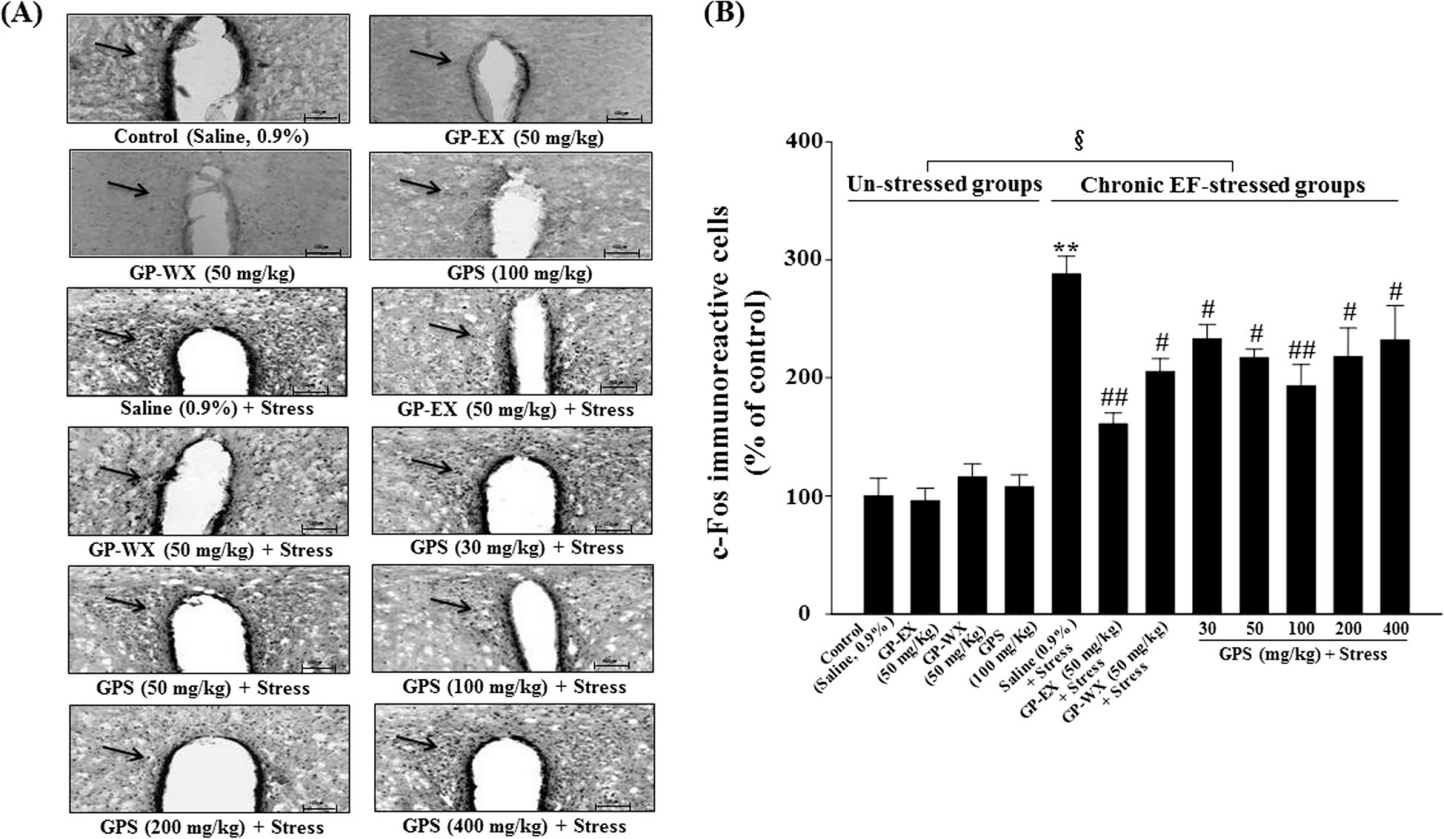
Representative photographs illustrating the effects of GPS on c-Fos-immunoreactive cells in the PVN (**a**) and the number of c-Fos-immunoreactive cells in the PVN (**b**). Mice (ICR, male, 25–30 g) were orally treated with GPS (30, 50, 100, 200 and 400 mg/kg), GP-WX (50 mg/kg), GP-EX (50 mg/kg, positive control) or saline (0.9 %) once a day for 10 days. Mice were also exposed to EF stimuli (intensity, 0.6 mA, 1 s every 5 s, periods, 3 min) for chronic stress for 10 days. GPS (100 mg/kg) was shown as a representative of the un-stressed groups. **a** Immunoblots of lysates from the brain were probed with c-Fos antibodies, and the total c-Fos-immunoreactive cells were measured as described under the Methods section. The arrow indicates nucleus of c-Fos-positive neurons. Scale bar is 100 μm. **b** The number of c-Fos-immunoreactive cells was counted in the PVN and was expressed as a percentage of the control groups. The number of c-Fos-immunoreactive cells of the control groups was 24 ± 4 cells per section. The results are expressed as means ± S.E.M. for 8 animals per group. **p < 0.01 compared with control group; ^#^ *p* < 0.05, ^##^ *p* < 0.01 compared with chronic EF-stressed group (one-way ANOVA followed by Tukey’s test); ^§^ *p* < 0.05 compared with between the un-stressed groups and stressed groups (two-way ANOVA followed by Tukey’s test)

GPS (30–400 mg/kg) for 10 days did not alter the expression of c-Fos (GPS at 100 mg/kg was shown as a representative) (Fig. 4). The expression of c-Fos protein in the PVN markedly increased to 188 % (*p* < 0.01) by exposure to chronic EF stress for 10 days, compared with control group (Fig. 4a and b). However, c-Fos expression was reduced by treatment with GPS (50–400 mg/kg) for 10 days in the PVN (Fig. 4a). The number of c-Fos-immunoreactive cells in the PVN, which increased by chronic EF stress, significantly decreased by 19.4 % (*p* < 0.05), 23.6 (p <0.05), 30.2 % (*p* < 0.01), 24.3 % (*p* < 0.05) and 24.0 % (*p* < 0.05), respectively by treatment with GPS (30, 50, 100, 200 and 400 mg/kg), compared with chronic EF-stressed group (Fig. 4b). c-Fos expression of the stressed groups also significantly increased compared with the un-stressed groups (F = 23.192, *p* < 0.05) (Fig. 4b).

In addition, cFos expression was reduced by treatment with GP-WX (50 mg/kg) for 10 days in the PVN (Fig. 4a) and the increased number of c-Fos-immunoreactive cells by chronic EF stress was significantly reduced by 28.7 % (*p* < 0.05) by treatment with GP-WX (50 mg/kg), compared with chronic EF-stressed group (Fig. 4b).

## Discussion

Chronic stress-induced anxiety disorders are currently one of the most important public health concerns. Recently, GP-EX has been reported to have an ameliorating effect on chronic EF stress-induced anxiety disorders through the elevated plus-maze and marble burying tests in mice [6]. In this study, the anxiolytic effects of the gypenoside-enriched components, GPS, and GP-WX on chronic EF stress-induced anxiety disorders were investigated. In addition, their anxiolytic functions were compared with those by GP-EX as a positive control [6], in order to investigate whether GPS was the main functional components for anxiety disorders.

The anxiety behavior in the elevated plus-maze test is expressed as a passive avoidance in response to a potential threat. In contrast, the defensive burying behavior in the marble burying test represents an active coping strategy in response to a discrete threat [20]. In addition, spontaneous locomotor activity is reduced in response to anxiety disorders and sedation [9].

GPS (100–400 mg/kg) has beneficial effects on mouse model of PD [12, 13]. Therefore, GPS at the ranges of 30–400 mg/kg was selected to examine the ameliorating effects on chronic stress-induced anxiety disorders in mice. In our study, treatment with GPS at 100–200 mg/kg significantly recovered chronic EF stress-reduced the number of open arm entries and the time spent on open arms (Fig. 1a and b). Closed arm entries, which are a measure of motor activity in elevated plus-maze, were slightly decreased by GPS, but it was not significant (data not shown). The ratios of the number of open arm entries to the total arm entries, which is considered to be related to the level of anxiety, were significantly reduced in the chronic EF-stressed groups. GPS (100–400 mg/kg) reduced the number of marbles buried which was increased by chronic EF stress (Fig. 2). In addition, the spontaneouse locomotor activity is related to dopamine levels [21]. At early periods of 2–3 days, the locomotor activities of control group were increased by acute stress, which was caused by increasing dopamine levels [22]. However, at later periods of 6–10 days, anxiety and/or depression were induced by the repeated chronic stress, and in these states, the spontaneous locomotor activities were gradually decreased below than control group [6]. In this study, GPS (100–200 mg/kg) reversed spontaneous locomotor activity for day-6 to day-10. GPS also showed the significant protective effects between the un-stressed groups and stressed groups at day-10, compared with day-1. These results indicate that GPS shows the anxiolytic functions without decreasing spontaneous locomotor activity in mice. In addition, treatment with GP-WX (50 mg/kg) exhibited an ameliorating effect on chronic EF stress-induced anxiety disorders which was respectively measured by elevated plus-maze, marble burying and locomotor activity (Figs. 1, 2 and 3).

Chronic stress-induced anxiety disorders decreases dopamine and serotonin levels in the brain [2]. Chronic stress-induced anxiety-like states maintains the increased levels of corticosterone compared with the un-stressed states. However, the levels of corticosterone induced by chronic stress are lower than those by acute stressed states and these reduced levels of corticosterone lead to regulate dopamine and serotonin levels [2]. In addition, anxiolytic-like behavioral responses in the elevated plus-maze test are mediated by the serotonin receptor functions [5]. The number of marbles buried is reduced by benzodiazepine receptor agonist (diazepam) and serotonin reuptake inhibitor (fluvoxamine) [6]. Dopamine receptor blocking agents inhibit spontaneous locomotor activity [21]. In this study, dopamine and serotonin levels were reduced by chronic EF stress in the brain, and they were increased by treatment with GPS (100–200 mg/kg) (Table 1). In contrast, the serum levels of corticosterone obviously increased by chronic EF stress, and they were also reversed by treatment with GPS (100–200 mg/kg) (Table 1). These results suggest that GPS can effectively ameliorate chronic stress-induced anxiety disorders by modulating the levels of dopamine, serotonin and corticosterone. In addition, the same patterns were also obtained by treatment with GP-WX (50 mg/kg) (Table 1). The serum levels of corticosterone induced by chronic EF stress were also modulated by treatment with GP-WX (50 mg/kg) (Table 1).

Among the Fos family members, FosB levels increase after being exposed to chronic stress [23], and the c-Fos expression in the PVN regions increases by chronic and acute stress in mice and rats [3, 4]. In addition, the PVN is essential for regulation of the HPA axis and may influence HPA axis hormone secretion such as corticosterone [24, 25]. GPS (30–400 mg/kg) significantly reduced c-Fos expression induced by chronic EF stress (Fig. 4), and also reduced the serum levels of corticosterone (Table 1). These results suggest that the modulation of c-Fos expression by GPS plays a role in the protective function in anxiety disorders induced by chronic EF stress. Chronic EF stress-induced c-Fos expression also decreased by GP-WX (50 mg/kg) in the PVN regions (Fig. 4).

In this study, GPS (30 and 50 mg/kg) exhibited the trend of anxiolytic effects on chronic EF stress-induced anxiety disorders, but it was not significant. GPS (400 mg/kg) also showed the anxiolytic effects on chronic EF stress-induced anxiety disorders. However, GPS at the ranges of 100–200 mg/kg showed a maximal efficacy on anxiolytic functions, as compared with GPS (30, 50 and 400 mg/kg). The high dose of GPS (400 mg/kg) might not be adopted for an oral administration in mice and the similar results have been also observed in a previous study [13].

GPS has been found to protect aortic endothelial cells against oxidative damage [26]. GPS shows the protective effects on oxidative stress induced by glutamate-induced neurotoxicity [27]. In addition, oxidative stress may cause the anxiety-related behaviors and the antioxidants could be used for the prevention or reduction of the high anxiety [28]. It is therefore possible to explain that the symptoms of anxiety disorders induced by chronic EF stress can be relieved by treatment with GPS and GP-WX, which has been closely mediated by the anti-oxidative activity in rodents.

In the present study, GPS at 100–200 mg/kg, but not 50 mg/kg, showed an equivalent efficacy on the anxiety behavioral tests, as compared with GP-EX at 50 mg/kg. However, GP-WX (50 mg/kg) showed a slightly less effect on the chronic stress-induced anxiety disorders than GP-EX (50 mg/kg) and GPS (100–200 mg/kg). GP-WX or GP-EX has been identified to have many kinds of GPS, flavonoids, polysaccharides, vitamins and amino acids [10]. GPS, GP-WX and GP-EX showed the different chromatogram patterns by the levels of bioactive components (the supplement data): GP-EX contained mainly the gypenoside, flavonoid and polysaccharide components, and GP-WX also contained the polysaccharide components. The levels of gypenoside components in GPS were higher than those in GP-EX. In addition, the levels of polysaccharide components in GP-EX were higher than those in GP-WX, which was much higher than those in GPS. The polysaccharides of GP-EX show various functions, including anti-aging, anti-fatigue, improving-immune competence and potential anti-oxidant [29, 30]. Polysaccharides supplementation also has beneficial effects on attenuating the oxidative stress induced by exhaustive exercise in rats [31]. These results suggest that GPS plays an important role in anti-anxiety disorders, and that besides GPS, the bioactive components including flavonoids and polysaccharides in GP-EX or GP-WX also show the anxiolytic-like functions. However, the intensities of anxiolytic-like functions between GPS and polysaccharides from GP need to be studied.

GPS (50–400 mg/kg) for 10 days does not exhibit the adverse effects, such as weight loss, diarrhea, vomiting and death. The values of LD_50_ of total GPS are 755–838 mg/kg (injected into the abdominal cavity) and 402 mg/kg (±18.2 mg/kg, i.p.) in mice [32]. GP-WX (750 mg/kg) does not also produce any significant toxicity in rats during 6-month period of treatment [33]. The lethal dose of total GP-EX is 1800–2000 mg/kg (p.o.) (data not shown). These results suggest that GPS might be safe more than GP-EX although GPS shows a less efficacy on chronic stress-induced anxiety disorders than GP-EX. However, GPS, GP-WX and GP-EX have been proved as safe herbal materials for treatment with animal models [6, 10].

## Conclusion

GPS (100–200 mg/kg) exhibited the anxiolytic effects on chronic EF stress-induced anxiety behaviors in mice, which was evaluated by the elevated plus-maze and marble burying and spontaneous locomotor activity tests by modulating the levels of dopamine, serotonin, corticosterone and c-Fos expression. GP-WX also showed the anxiolytic activity on chronic EF stress-induced anxiety behaviors in mice, but it was lower activities than those obtained by GPS. GPS may serve as a phytonutrient in chronic stress-induced anxiety disorders and clinical tests of GPS need to be conducted further.

### Availability of data and materials 

Not applicable.

## Abbreviations

GPS: Gypenosides
EF stress: Electric footshock stress
PVN: Paraventricular nuclei
GP: *Gynostemma pentaphyllum*
GP-EX: Ethanol extract from GP
GP-WX: Water extract from GP
PD: Parkinson’s disease
HIAA: 5-Hydroxyindoleacetic acid
